# The impact of dance/movement psychotherapy on social skills in individuals with autism spectrum disorder: a meta-analysis

**DOI:** 10.3389/fpsyg.2026.1768543

**Published:** 2026-08-06

**Authors:** Sung Hee Koh

**Affiliations:** Department of Physical Education, Graduate School of Education, Myongji University, Seoul, Republic of Korea

**Keywords:** autism spectrum disorder, dance/movement psychotherapy, meta-analysis, social skills, social skills in individuals with autism spectrum disorder

## Abstract

**Purpose:**

This study conducted a meta-analysis to evaluate the effectiveness of Dance/Movement Psychotherapy (DMP) on social skills in individuals with Autism Spectrum Disorder (ASD). Although DMP has been widely applied as an embodied and relational intervention, quantitative evidence regarding its impact on social functioning remains limited.

**Methods:**

A systematic literature search was performed across major databases (PubMed and PsycNet) to identify studies published between 2000 and 2025. Eligible studies examined DMP-based interventions targeting social skills in individuals with ASD and included a comparison group. Four studies (*k* = 4) with a total of 93 participants met the inclusion criteria. Effect sizes were calculated using a random-effects model, and heterogeneity and publication bias were assessed using standard statistical procedures.

**Results:**

The meta-analysis revealed a significant overall effect of DMP on social skills (Hedges’ g = −0.97, 95% CI: −1.69 to −0.25), indicating substantial improvement compared with control conditions. Because higher scores on commonly used measures such as the Social Responsiveness Scale reflect greater social impairment, negative effect sizes signify enhanced social skills. Moderate heterogeneity was observed across studies (*I*^2^ = 58.30%), and publication bias analyses suggested limited stability of the pooled effect.

**Conclusion:**

The findings provide preliminary quantitative evidence suggesting that DMP may contribute to improvements in social skill-related outcomes in individuals with ASD. However, the small number of available studies and indications of publication bias warrant cautious interpretation. Further high-quality randomized studies with standardized outcome measures are needed to strengthen the evidence base for DMP in ASD interventions.

## Introduction

Autism Spectrum Disorder (ASD) is a lifelong neurodevelopmental condition characterized by persistent difficulties in social communication and interaction, alongside restricted and repetitive behavioral patterns (American Psychiatric Association, 2013). Since ASD was first systematically examined in the 1960s—with an estimated prevalence of 0.04%—global rates have risen dramatically, reaching approximately 1%–2% today, or an estimated 78 million individuals worldwide (Baxter et al., 2015; Maenner et al., 2023). This sharp increase has intensified public health concerns regarding both the causes of ASD and the development of effective interventions. Core diagnostic features of ASD primarily involve impairments in social communication and interaction, as well as restricted and repetitive behaviors. In addition, many individuals with ASD experience motor-related difficulties and atypical movement patterns, which may further affect adaptive functioning and everyday social participation (Kunzi, 2015).

In the present study, the term “social skills” is used as a multidimensional construct encompassing social communication, interpersonal engagement, emotional reciprocity, social responsiveness, and adaptive social interaction. Individuals with ASD commonly experience difficulties across these domains, including challenges in interpreting social cues, maintaining reciprocal interactions, understanding emotions and facial expressions, and responding appropriately within social contexts (Watkins et al., 2017). Such impairments often hinder the formation of meaningful interpersonal relationships and may contribute to increased risks of social isolation, anxiety, and other mental health difficulties.

In addition to social-communication challenges, many individuals with ASD exhibit motor-related symptoms and atypical movement patterns. These may include stereotyped and repetitive behaviors such as hand flapping, body swaying, or fixed facial expressions, which frequently intensify during situations involving stress, excitement, or sensory overload (Mackenzie, 2018; Melo et al., 2020). Because social, emotional, sensory, and motor experiences are often closely interconnected in ASD, these difficulties may collectively influence developmental trajectories, adaptive functioning, and everyday social participation. Consequently, ASD can place substantial emotional, psychological, and economic burdens on families and caregivers responsible for long-term support and supervision (Karst and Van Hecke, 2012; McIntyre et al., 2023).

Given that ASD is lifelong and currently incurable, research has increasingly focused on identifying interventions that can meaningfully improve social and behavioral functioning. Existing approaches generally fall into two categories: pharmacological treatment and behavioral intervention. Psychotropic medications may reduce anxiety, repetitive behaviors, or attentional difficulties but can also produce adverse effects such as drowsiness or appetite suppression, and their efficacy varies across individuals (Sharma et al., 2018). Behavioral interventions—including Applied Behavior Analysis, the Denver Early Start Model, sensory integration therapy, and DIR/Floortime—have demonstrated effectiveness but are often highly structured, resource-intensive, and limited in their adaptability to diverse environments (Deng et al., 2023; Jayousi et al., 2023; Rossilli, 2021).

Dance/Movement Psychotherapy (DMP) offers a distinctive therapeutic approach grounded in embodied, relational, and non-verbal processes. Because many individuals with ASD experience challenges in verbal communication and interpersonal reciprocity, movement-based and body-oriented interventions may provide alternative pathways for social engagement and emotional expression. Through mirroring, rhythmic synchronization, embodied attunement, and shared movement experiences, DMP aims to facilitate interpersonal connection and enhance social responsiveness in ways that may be particularly meaningful for individuals with ASD.

From a social and embodied perspective, DMP may facilitate interpersonal attunement, attention regulation, and non-verbal social engagement through coordinated movement experiences (Koch et al., 2015). Nonetheless, empirical findings remain mixed. Some studies report significant gains in social functioning, whereas others indicate no measurable improvement. For instance, Mastrominico et al. (2018) found that a 10-week manualized DMP intervention did not significantly improve socio-emotional abilities in adults with ASD. Similarly, Walsh (2024) reported no observable enhancement in social skills among children and adolescents receiving DMP. These inconsistencies complicate the interpretation of DMP as a reliable intervention for social functioning and social skill-related outcomes in individuals with ASD and underscore the need for a systematic evaluation.

A meta-analytic approach can address these discrepancies by quantitatively synthesizing available evidence and assessing the methodological rigor of existing studies. Previous meta-analyses of physical activity interventions have highlighted potential benefits for ASD populations, including improvements in motor skills, social functioning, and emotional regulation (Healy et al., 2018; Sowa and Meulenbroek, 2012). However, research specifically examining DMP for social skill enhancement remains limited, and questions persist regarding optimal intervention duration, session structure, and therapeutic components.

Therefore, this study aims to provide a preliminary quantitative synthesis of the available evidence regarding the effects of DMP on social skill-related outcomes in individuals with ASD. By synthesizing the currently available studies, this review seeks to contribute to the emerging evidence base for embodied and movement-based interventions in ASD.

## Methods

This meta-analysis was conducted in accordance with the Preferred Reporting Items for Systematic Reviews and Meta-Analyses (PRISMA) guidelines and followed established procedures for the transparent reporting of systematic reviews (Page et al., 2021). A formal review protocol was not preregistered in PROSPERO or other systematic review registries.

### Selection of studies for inclusion

For this meta-analysis, the literature selection focused on research studies examining the effectiveness of Dance/Movement Psychotherapy (DMP) on social skill-related outcomes in individuals with Autism Spectrum Disorder (ASD). The selection process followed systematic review procedures in accordance with PRISMA guidelines and incorporated computerized database searches as well as manual snowball searches of reference lists from relevant review articles and included studies.

Studies were included if they: (1) involved participants formally diagnosed with ASD; (2) evaluated a DMP intervention or movement-based psychotherapeutic approach; (3) included a comparison or control condition; (4) quantitatively assessed social skill-related outcomes; (5) provided sufficient statistical information to calculate effect sizes; and (6) were published in English between 2000 and 2025. Studies were excluded if they: (1) did not include a control or comparison group; (2) focused primarily on recreational dance, physical exercise, or general movement activities without psychotherapeutic components; (3) lacked quantitative outcome data; or (4) consisted of review articles, conference abstracts, editorials, or theoretical papers.

The computerized literature search was conducted across major academic databases, including PubMed and PsycNet. The search targeted studies published between 2000 and 2025. The following search string was used to identify potentially eligible studies: (“dance movement therapy” OR “dance/movement psychotherapy” OR “movement psychotherapy” OR “movement therapy” OR “authentic movement” OR “primitive expression”) AND (“social skills” OR “social functioning” OR “social responsiveness” OR “social communication”) AND (“autism spectrum disorder” OR “ASD” OR “Asperger” OR “childhood disintegrative disorder” OR “neurodevelopmental disorder” OR “pervasive developmental disorder”).

Reference lists of relevant review papers and included studies were manually screened to identify additional eligible articles. Grey literature, conference proceedings, and unpublished studies were not systematically searched.

Two independent reviewers screened studies and extracted the following data from each eligible article: study design, participant characteristics, total sample size, gender, age, intervention duration, outcome measures, and statistical data required for effect size calculation. The accuracy of the extracted data was verified by comparing coding sheets between the two reviewers, and any disagreements were resolved through discussion and consensus.

### DMP interventions

DMP interventions in the included studies were defined as structured movement-based therapeutic approaches designed to enhance social engagement and nonverbal communication in individuals with ASD. Across studies, interventions were delivered by trained DMP therapists or clinicians with equivalent expertise. Core therapeutic components commonly included mirroring, embodied attunement, rhythmic coordination, and guided improvisational movement aimed at facilitating interpersonal responsiveness. Although specific session formats varied, all interventions emphasized movement as the primary medium for supporting social interaction. For coding purposes, interventions were categorized based on session format, facilitator qualifications, intervention duration, and primary therapeutic techniques. Only programs explicitly grounded in DMP principles or movement-based psychotherapeutic methods were included.

### Dependent variable

The dependent variable in this meta-analysis consisted of social skill-related outcomes associated with social functioning in individuals with ASD. Because the included studies assessed heterogeneous but conceptually related domains—including social responsiveness, interpersonal engagement, and communication-related behaviors—the outcomes were synthesized under a broader social functioning framework. These domains were quantitatively assessed using standardized instruments such as the Social Responsiveness Scale (SRS). Due to variability in the operationalization of social skill-related constructs across studies, outcome measures differed among the included studies.

### Coding methodology

Following established norms for meta-analytic research, we meticulously designed our coding procedure to thoughtfully capture and quantify crucial study characteristics and outcomes. In our thorough coding process, we systematically extracted several key study characteristics from each study. This encompassed details such as authorship, year of publication, study setting, study design, length of intervention, gender, mean age of participant group, number of participants in experimental and control groups, and means and standard deviations of intervention effectiveness at pretest and posttest.

### Effect size calculations and statistical analysis

For this meta-analysis, Hedges’ g was used as the standardized effect size metric because it provides a bias-corrected estimate appropriate for studies with relatively small sample sizes. Effect sizes were calculated using post-intervention means and standard deviations from the experimental and control groups. When necessary, effect sizes were directionally adjusted so that negative values consistently reflected improvements in social impairment symptoms and social skill-related outcomes. Each effect size was weighted by the inverse of its variance before pooling across studies. The overall pooled effect size and corresponding variance were calculated according to the procedures described by Hedges and Olkin (1985). In interpreting effect sizes, values of approximately 0.2, 0.5, and 0.8 were considered small, medium, and large effects, respectively, following Cohen’s conventional criteria (Cohen, 1988).

Because variability across studies was anticipated due to differences in participant characteristics, intervention protocols, and outcome measures, statistical heterogeneity was assessed using Cochran’s Q statistic and the I^2^ index (Higgins and Thompson, 2002). When substantial heterogeneity was identified (*p* < 0.10 or I^2^ > 50%), a random-effects model was applied in accordance with the recommendations of Higgins et al. (2003). Given the methodological diversity among included studies, the random-effects model was considered the most appropriate approach for estimating the pooled intervention effect.

Publication bias was evaluated using multiple complementary methods, including visual inspection of funnel plot asymmetry, Egger’s regression test, Begg’s rank correlation test, and Duval and Tweedie’s (2000) trim-and-fill procedure. Statistical significance was set at *p* < 0.05 for all analyses. All statistical analyses were conducted using the ‘meta’ and “metafor” packages in R version 4.2.2.

## Results

### Study selection

After conducting the database search, a total of 31 records were initially identified, and an additional 5 records were identified through manual snowballing procedures. Following duplicate removal, 28 articles remained for title and abstract screening. Thirteen studies were excluded at this stage because they were unrelated to ASD, did not involve DMP interventions, or did not assess social skill-related outcomes.

Full-text eligibility assessment was subsequently conducted for eight articles considered potentially relevant to the effects of DMP on social skills in individuals with ASD. Of these, four studies were excluded following full-text review. Three studies were excluded because they did not provide sufficient quantitative data required for effect size calculation, and one additional study was excluded because the intervention did not meet the operational definition of DMP established for the present review, including interventions primarily based on recreational dance or general movement activities without psychotherapeutic components.

**Figure 1:**
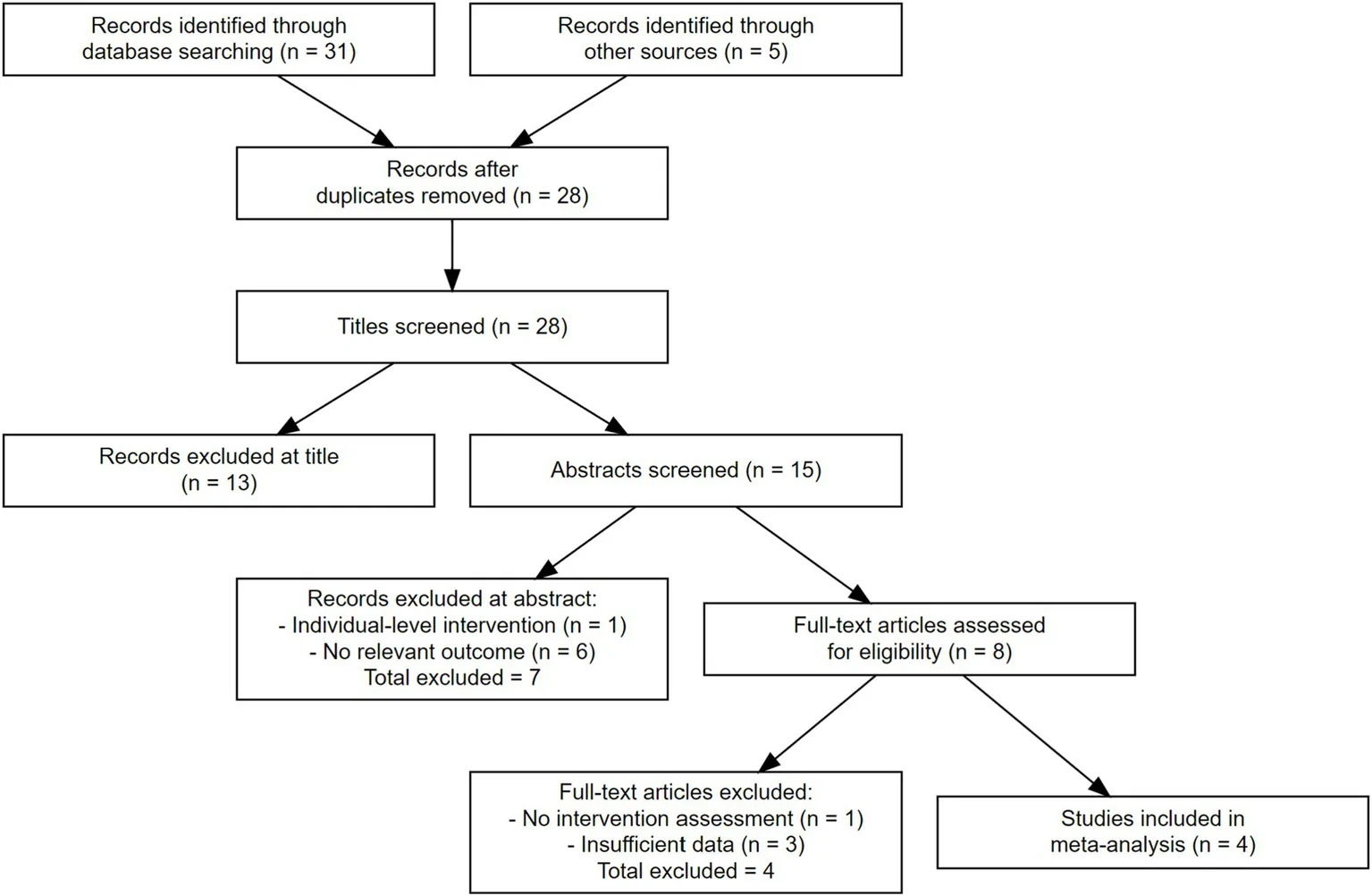
Flowchart of the study selection process according to the PRISMA protocol declarations.

Ultimately, four studies met all eligibility criteria and were included in the final meta-analysis (see Figure 1). Detailed characteristics of the included studies are presented in Table 1.

**Table 1:** Characteristics of included studies.

Study	Design	Participants	DMP intervention	Duration	Comparator	Outcome measure
Koch et al. (2015)	Quasi-experimental control study	Young adults with ASD (*n* = 31)	Manualized DMP intervention emphasizing mirroring, embodied attunement, and movement interaction	10 weeks	Control condition	Social Responsiveness Scale (SRS)
Bergmann et al. (2021)	Quasi-randomized partly controlled study	Adults with ASD and intellectual disability	Autism-Competency-Group movement-based psychotherapeutic intervention	Not clearly specified	Waitlist control group	Social functioning outcomes
Ren et al. (2022)	RCT	Children with ASD	Dance movement and music mixed treatment in parent–child setting	Not clearly specified	Control group	Social communication impairment measures
Cui and Wang (2024)	RCT	Children with ASD	Dance therapy intervention targeting social communication ability	Not clearly specified	Control group	Social communication ability measures

### Assessment of risk of bias

The risk of bias was evaluated separately for randomized and non-randomized studies. Randomized controlled trials were assessed using the revised Cochrane Risk of Bias Tool (RoB 2), whereas non-randomized intervention studies were evaluated using the ROBINS-I tool. Because these instruments differ substantially in both conceptual framework and assessment domains, the findings were reported separately rather than combined into a single graphical summary.

Among the randomized controlled trials included in this review (Cui and Wang, 2024; Ren et al., 2022), the overall risk of bias was judged as presenting some concerns rather than high risk. Although both studies reported random allocation procedures and demonstrated relatively low risk regarding missing outcome data and selective reporting, limited methodological detail regarding allocation concealment and outcome assessment procedures introduced some uncertainty, particularly within the domains of randomization and measurement of outcomes.

In contrast, the non-randomized studies (Bergmann et al., 2021; Koch et al., 2015) demonstrated greater methodological variability and generally higher risks of bias. The primary concerns involved potential confounding, non-random group allocation, and participant selection bias, which are inherent limitations of quasi-experimental designs. Additional concerns related to outcome measurement bias were identified in studies relying heavily on self-report measures or non-blinded assessments. Overall, Koch et al. (2015) was judged to have a moderate risk of bias, whereas Bergmann et al. (2021) demonstrated a more serious overall risk due to quasi-randomized allocation procedures and the use of partly self-developed outcome measures.

Taken together, these findings indicate that the methodological rigor of the included studies varied considerably, with randomized controlled trials generally providing stronger internal validity than non-randomized studies. Accordingly, although the pooled findings suggest that DMP interventions may contribute to improvements in social skill-related outcomes in individuals with ASD, the overall evidence base should still be interpreted cautiously, given the limited number of rigorously controlled studies.

Detailed domain-level risk-of-bias assessments for randomized controlled trials and non-randomized studies are presented in Tables 2, 3, respectively.

**Table 2:** Risk-of-bias assessment for randomized controlled trials (RoB 2).

Study	Randomization process	Deviations from intended interventions	Missing outcome data	Measurement of the outcome	Selection of the reported result	Overall risk of bias
Ren et al. (2022)	Some concerns	Low risk	Low risk	Some concerns	Low risk	Some concerns
Cui and Wang (2024)	Some concerns	Some concerns	Low risk	Some concerns	Low risk	Some concerns

**Table 3:** Risk-of-Bias assessment for non-randomized and quasi-experimental studies (ROBINS-I).

Study	Confounding	Participant selection	Classification of intervention	Deviations from intended interventions	Missing data	Measurement of outcomes	Selection of reported results	Overall risk of bias
Koch et al. (2015)	Moderate	Moderate	Low	Moderate	Low	Moderate	Low	Moderate
Bergmann et al. (2021)	Serious	Moderate	Low	Moderate	Low	Moderate	Low	Serious

### Social skills

A total of four studies (*k* = 4) involving 93 participants (47 in the experimental groups and 46 in the control groups) were included in the meta-analysis. The random-effects model yielded a statistically significant pooled effect size (Hedges’ g = −0.97, 95% CI: −1.69 to −0.25). This finding suggests that DMP interventions may be associated with improvements in social skill-related outcomes in individuals with ASD compared with control conditions. However, the findings should be interpreted cautiously given the small number of included studies, relatively small sample sizes, methodological heterogeneity, and inclusion of both randomized and non-randomized study designs. The overall effect was statistically significant (z = −2.64, *p* = 0.0083). Because higher scores on commonly used measures such as the Social Responsiveness Scale reflect greater social impairment, negative effect sizes in the present analysis indicate improvements in social skill-related outcomes.

**Figure 2:**
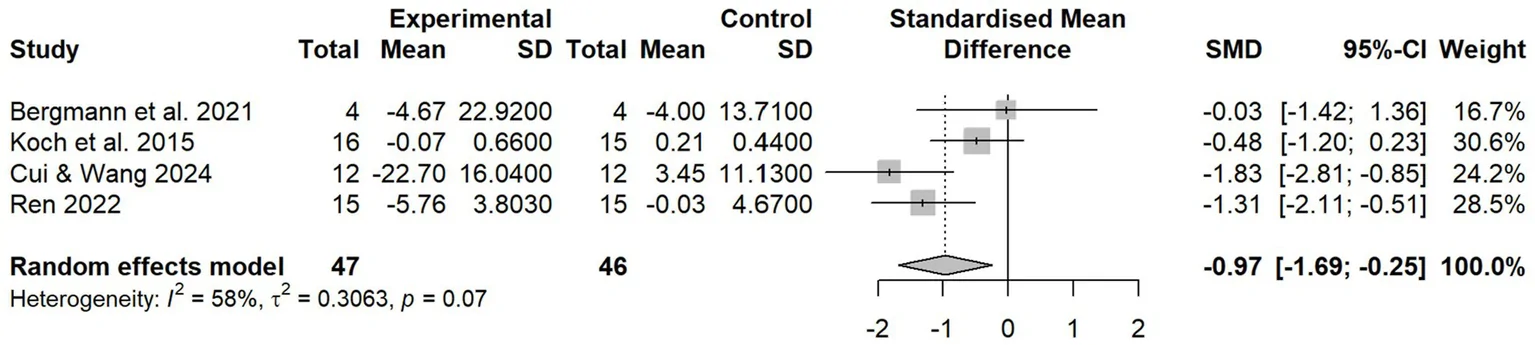
Forest plot of meta-analysis for DMP on social skills in individuals with ASD.

Moderate heterogeneity was observed across studies, with an *I*^2^ value of 58.3% (H = 1.55), indicating that approximately half of the variability in effect sizes was attributable to true differences rather than sampling error. Although the Q-test for heterogeneity did not reach conventional significance [Q(3) = 7.19, *p* = 0.066], the magnitude of *I*^2^ suggests moderate between-study variability. The tau^2^ estimate was 0.31, derived using the restricted maximum likelihood estimator, with a broad confidence interval reflecting some uncertainty due to the small number of studies. Overall, these findings demonstrate a significant pooled effect under the random-effects model while acknowledging moderate heterogeneity among the included studies. The forest plot illustrating the individual and pooled effect sizes is presented in Figure 2.

The observed heterogeneity may partly reflect differences across studies in participant age groups, intervention formats, therapeutic structure, intervention duration, and outcome measurement approaches. For example, the included studies involved both children and adults with ASD and incorporated varying forms of DMP interventions, ranging from structured manualized protocols to mixed movement-based therapeutic approaches. In addition, outcome measures differed across studies, including assessments of social responsiveness, interpersonal functioning, and social communication-related behaviors. Because only four studies were eligible for inclusion, formal subgroup analyses or meta-regression could not be conducted reliably. Therefore, potential sources of heterogeneity were explored descriptively rather than statistically.

### Publication bias

Publication bias was assessed using visual inspection of the funnel plot, Egger’s regression test, Begg’s rank correlation test, and Duval and Tweedie’s (2000) trim-and-fill procedure. However, because only four studies were included, these procedures have limited inferential value and should be interpreted cautiously.

**Figure 3:**
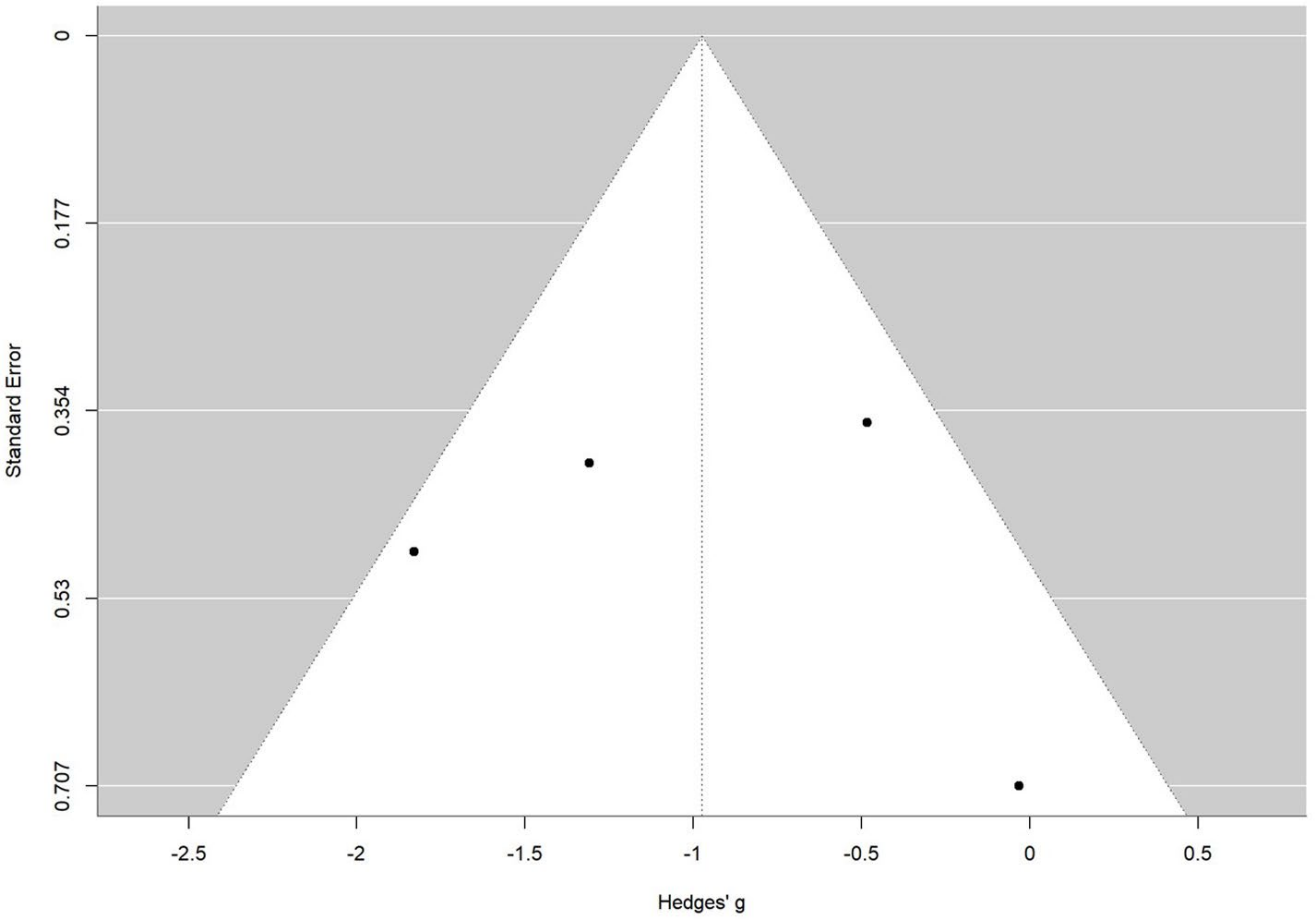
Funnel plot of observed studies assessing the effects of DMP on social skills in individuals with ASD.

**Figure 4:**
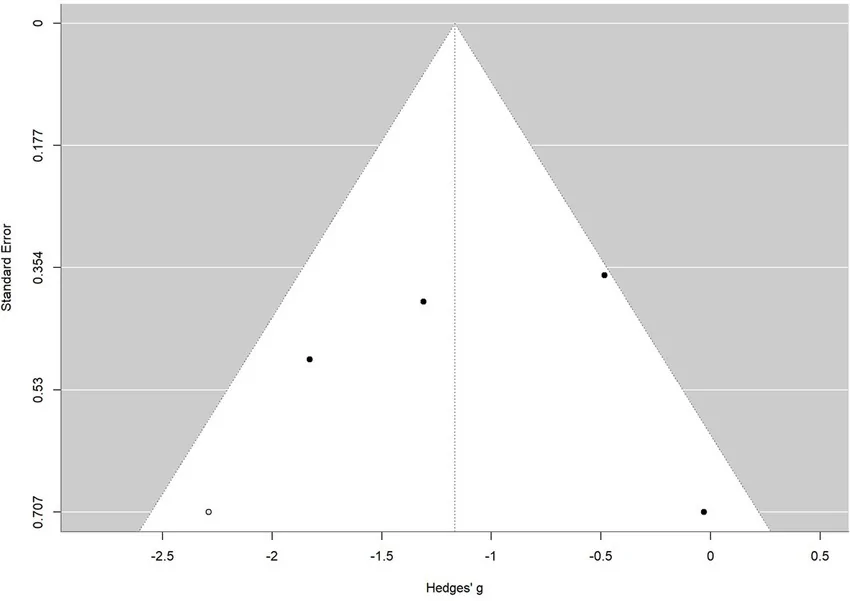
Trim-and-fill–adjusted funnel plot for the DMP meta-analysis on social skills.

Visual inspection of the funnel plot suggested slight asymmetry, although Egger’s regression test (t = 0.16, df = 2, *p* = 0.889) and Begg’s rank correlation test (*p* > 0.05) did not indicate statistically significant evidence of publication bias. The funnel plot is presented in Figure 3. The trim-and-fill procedure estimated one potentially missing study on the left side of the funnel plot. The trim-and-fill–adjusted funnel plot is shown in Figure 4. Nevertheless, given the very small number of included studies, these findings remain exploratory and do not permit firm conclusions regarding publication bias. A summary of the publication bias diagnostic procedures and findings is presented in Table 4.

**Table 4:** Summary of publication bias diagnostics for the meta-analysis.

Method	Results	Implications
Funnel plot (Visual Inspection)	Slight asymmetry observed	Possible indication of publication bias, though subjective and limited by small k
Egger’s Regression Test	*t* = 0.16, df = 2, *p* = 0.889	No statistically significant asymmetry; no evidence of small-study effects
Begg’s Rank Correlation Test	*p* > 0.05	No significant rank correlation; no evidence of publication bias
Trim-and-Fill (Duval and Tweedie, 2000)	1 potentially missing study imputed,	Suggests possible asymmetry, although interpretation is limited by the very small number of included studies

## Discussion

This meta-analysis synthesized the current empirical evidence on the effectiveness of DMP in improving social skill-related outcomes among individuals with ASD. Across the included studies, DMP interventions were associated with improvements in social skill-related outcomes, with the pooled effect size suggesting a moderate-to-large effect following intervention. These findings are generally consistent with theoretical perspectives emphasizing embodied communication, kinesthetic attunement, and nonverbal synchrony as mechanisms that may enhance social engagement in ASD (Levy, 1988; Meekums, 2002; Payne, 2003; Karkou and Sanderson, 2006; Wengrower, 2010). Despite the considerable variability across studies, the overall pattern suggests the potential relevance of DMP as a complementary psychosocial intervention.

Although the included studies varied considerably in intervention structure, duration, and therapeutic format, several studies reported improvements in social skill-related outcomes following DMP interventions (Adler, 1968; Siegel, 1973; Erfer, 1995; Loman, 1995; Parteli, 1995; Torrance, 2003; Tortora, 2005, 2010; Martin, 2014; Scharoun et al., 2014). However, because the present meta-analysis did not directly compare intervention components or conduct subgroup analyses according to intervention format, no firm conclusions can be drawn regarding which specific therapeutic elements may have contributed to the observed effects. Components frequently discussed within the DMP literature—such as mirroring, rhythmic movement, embodied attunement, and non-verbal interpersonal engagement—remain theoretically relevant and may represent potential mechanisms requiring further empirical investigation in future studies (Devereaux, 2012; Howells et al., 2020).

At the same time, the findings should be interpreted cautiously due to several methodological and conceptual limitations. First, moderate heterogeneity was observed across studies, indicating considerable variability in intervention protocols, participant characteristics, and outcome measurement approaches. Importantly, the construct of “social skills” was operationalized inconsistently across studies. Some investigations relied primarily on standardized parent-report measures, whereas others incorporated therapist ratings or observational assessments. Such conceptual inconsistency complicates direct comparison across studies and may partly explain the variability observed in effect sizes. In addition, although the pooled effect size was statistically significant and fell within the moderate-to-large range according to conventional benchmarks, statistical significance does not necessarily imply clinically transformative improvement. The included studies employed heterogeneous outcome measures, differing sources of assessment, and variable definitions of social skill-related outcomes. Consequently, the observed pooled effect should not be interpreted as evidence that DMP produces uniformly meaningful functional changes across everyday social contexts. Rather, the findings should be viewed as preliminary evidence suggesting the potential for improvement in selected social and interpersonal domains that requires further validation through clinically rigorous and longitudinal research.

Beyond these methodological inconsistencies, it is also important to consider the fundamentally embodied and phenomenological nature of DMP itself (Association for Dance Movement Psychotherapy UK, 2020). Unlike highly structured behavioral interventions that primarily target observable outcomes, DMP is grounded in lived bodily experience, affective attunement, interpersonal movement dynamics, and non-verbal relational processes (Meekums, 2002; Payne, 2003; Karkou and Sanderson, 2006). Therapeutic change within DMP often emerges through subtle embodied experiences that may not be fully accessible through standardized quantitative assessment tools alone.

In particular, many therapeutic processes emphasized in DMP—including kinesthetic empathy, embodied synchrony, mirroring, affective resonance, and shared rhythmic engagement—are inherently difficult to operationalize using conventional social-skills scales (Levy, 1988; Loman, 1995; Devereaux, 2012). Standardized instruments such as the Social Responsiveness Scale primarily assess externally observable symptoms and behavioral impairments, but may insufficiently capture qualitative shifts in relational presence, emotional attunement, bodily awareness, or experiential connectedness that occur during movement-based therapeutic interactions.

From a phenomenological perspective, DMP conceptualizes the body not merely as a behavioral instrument but as a primary medium through which individuals experience, express, and negotiate relationships with others and the surrounding world (Meekums, 2002; Payne, 2003; Wengrower, 2010). This perspective may be particularly relevant for individuals with ASD, whose social and communicative experiences are often deeply intertwined with sensory, motor, and embodied forms of interaction (Tortora, 2005, 2010; Martin, 2014). Consequently, therapeutic improvements may initially emerge at pre-verbal, affective, or sensorimotor levels before becoming fully detectable through conventional behavioral assessments.

Furthermore, reliance solely on quantitative outcome aggregation may inadvertently reduce the complexity of DMP to narrowly measurable behavioral indicators. This may risk overlooking the process-oriented, relational, and experiential dimensions that are often considered central to therapeutic change within DMP practice (Levy, 1988; Karkou and Sanderson, 2006). Although standardized social-skills measures provide important clinical information, they may not adequately capture subtle transformations in embodied awareness, affective reciprocity, interpersonal presence, and non-verbal relational engagement that emerge throughout the therapeutic process. Nevertheless, standardized quantitative assessments still provide clinically meaningful indicators of observable social functioning and remain important for evaluating intervention-related changes across studies.

Accordingly, the findings of the present meta-analysis should be interpreted as preliminary quantitative evidence rather than a definitive evaluation of the full therapeutic complexity of DMP. Future research should therefore incorporate mixed-methods designs, qualitative interviews, observational movement analysis, phenomenological inquiry, and process-oriented methodologies in order to more comprehensively capture the embodied and relational dimensions of DMP interventions.

Another important limitation concerns the integration of randomized controlled trials (RCTs) and non-randomized intervention studies within the same meta-analytic model. Although combining both designs allowed for a broader synthesis of the limited available literature, non-randomized studies inherently carry higher risks of confounding, selection bias, and measurement bias. As a result, pooled effect sizes may overestimate intervention effectiveness. While separate risk-of-bias assessments were conducted using RoB2 and ROBINS-I, future meta-analyses would benefit from sensitivity analyses or subgroup comparisons restricted to rigorously controlled trials. Accordingly, the pooled effect reported in the present study should not be interpreted as definitive evidence of intervention efficacy, but rather as preliminary evidence that remains methodologically constrained and potentially influenced by study design limitations and bias within the non-randomized literature.

Publication bias also warrants careful consideration. Although Egger’s regression test and Begg’s rank correlation test did not reveal statistically significant asymmetry, the very small number of included studies substantially limits the statistical power of these procedures. In addition, the trim-and-fill analysis suggested the possible presence of one missing study, indicating that the current evidence base may not fully represent unpublished or null findings. Consequently, the magnitude of the pooled effect should be interpreted cautiously until a larger body of high-quality studies becomes available.

The small number of eligible studies represents one of the most significant limitations of this review. With only four studies meeting inclusion criteria, statistical precision is limited, and several meta-analytic procedures—including heterogeneity estimation and publication bias testing—yield less reliable results when the number of studies is fewer than 10. Additionally, the lack of long-term follow-up data across studies prevents conclusions about the durability of DMP-related improvements in social functioning. Given the developmental and lifelong nature of social skill difficulties in ASD, sustained gains may be particularly important to assess.

Despite these limitations, this review contributes meaningful insights to the emerging empirical foundation supporting DMP for ASD. It highlights the need for more rigorous, theory-driven trials with larger and more diverse samples, standardized outcome measures, and clear reporting of intervention components. Future research should also theoretically and empirically investigate potential mechanisms that have been proposed within the DMP literature—such as emotional attunement, body-awareness development, and sensorimotor integration—in order to better understand how movement-based therapeutic processes may relate to social skill-related outcomes in ASD (Erfer, 1995; Loman, 1995; Tortora, 2005, 2010; Martin, 2014; Howells et al., 2020). Because the present meta-analysis did not examine mediators, moderators, or mechanisms of change directly, these interpretations should be regarded as theoretical directions for future research rather than conclusions derived from the current findings. Comparative studies—including head-to-head evaluations with other psychosocial or movement-based interventions—may help determine whether DMP offers unique advantages or synergistic benefits (Nind and Hewett, 1988).

Overall, this meta-analysis provides promising preliminary evidence suggesting that DMP may contribute to improvements in social skill-related outcomes in individuals with ASD. However, the field remains in an early stage of empirical development, and robust conclusions will require a larger and more methodologically consistent body of research. Continued efforts to refine intervention protocols, strengthen methodological rigor, and expand the evidence base will be essential for strengthening the empirical foundation of DMP within ASD intervention research.

## Conclusion

This meta-analysis provides preliminary quantitative evidence suggesting that Dance Movement Psychotherapy (DMP) may contribute to improvements in social skill-related outcomes in individuals with Autism Spectrum Disorder (ASD). Across the four included studies, DMP interventions were associated with improvements in social skills compared with control conditions.

However, these findings should be interpreted cautiously. The small number of included studies, methodological heterogeneity, integration of randomized and non-randomized designs, variability in outcome measures, and potential publication bias substantially limit the strength and generalizability of the current evidence base. Moreover, statistically significant pooled effects do not necessarily indicate clinically transformative improvements across everyday social functioning.

Future research should prioritize rigorously designed randomized controlled trials with larger and more diverse samples, standardized outcome measures, transparent reporting practices, and long-term follow-up assessments. Additional studies are also needed to clarify the theoretical and therapeutic mechanisms underlying DMP interventions and to evaluate their clinical relevance across different ASD populations and intervention contexts.

## Data Availability

The data analyzed in this study were obtained from multiple previously published articles cited in the reference list. As this is a meta-analysis, there is no single repository or accession number. All datasets are publicly available through the original publications. Any further inquiries can be directed to the corresponding author.
